# Supplementary material for: A machine-learning method for biobank-scale genetic prediction of blood group antigens
Source: PLoS Comput Biol. 2024 Mar 21;20(3):e1011977. doi: 10.1371/journal.pcbi.1011977 (PMC10986993; doi:10.1371/journal.pcbi.1011977)
Supplement: S1 Text — Table A. Accuracy metrics for the Finnish random forest models in the Finnish test data set. Table B. Accuracy metrics for the Finnish random forest models in the Finnish train data set. Table C. Accuracy metrics for the Finnish random forest models in the Finnish full data set. Table D. Accuracy metrics for the Finnish gradient boosting models in the Finnish test data set. Table E. Accuracy metrics for the Finnish random forest models in the Danish full data set. Table F. Accuracy metrics for the Danish random forest models in the Danish test data set. Table G. Accuracy metrics for the Danish random forest models in the Danish train data set. Table H. Accuracy metrics for the Danish random forest models in the Danish full data set. Table I. Characteristics of the Danish random forest classification models. Table J. Blood group/HPA-1 genes and genetic regions. Fig A. Confusion matrices for the Finnish random forest models in the Finnish test data set. Fig B. Confusion matrices for the Finnish random forest models in the Finnish train data set. Fig C. Confusion matrices for the Finnish random forest models in the Finnish full data set. Fig D. Receiver operating characteristic and precision-recall curves for the Finnish random forest models in the Finnish test data se. Fig E. Posterior probability boxplots for the Finnish random forest models in the Finnish test data set. Fig F. Posterior probability boxplots for the Finnish random forest models in the Finnish train data set. Fig G. Posterior probability boxplots for the Finnish random forest models in the Finnish full data set. Fig H. Confusion matrices for the Finnish gradient boosting models in the Finnish test data set. Fig I. Posterior probability boxplots for the Finnish gradient boosting models in the Finnish test data set. Fig J. Confusion matrices for the Danish random forest models in the Danish train data set. Fig K. Confusion matrices for the Danish random forest models in the Danish test data set. Fig L. [file pcbi.1011977.s001.pdf]

A machine-learning method for biobank-scale genetic prediction of blood group antigens

SUPPLEMENTARY MATERIALS

Hyvärinen K[1*], Haimila K[2], Moslemi C[3,4], Blood Service Biobank[5], Olsson ML[6,7], Ostrowski SR[8,9], Pedersen OB[3,9], Erikstrup C[10], Partanen J[1], Ritari J[1]

1. *Research and Development, Finnish Red Cross Blood Service, Helsinki, Finland*
2. *Blood Group Unit, Finnish Red Cross Blood Service, Vantaa, Finland*
3. *Department of Clinical Immunology, Zealand University Hospital,* Køge, *Denmark*
4. *Department of Clinical Immunology, Aarhus University Hospital, Aarhus, Denmark*
5. *Finnish Red Cross Blood Service, Vantaa, Finland*
6. *Department of Laboratory Medicine, Lund University, Lund, Sweden*
7. *Department of Clinical Immunology and Transfusion Medicine, Office for Medical Services, Region Skåne, Sweden*
8. *Department of Clinical Immunology, Copenhagen University Hospital, Rigshospitalet, Copenhagen, Denmark*
9. *Department of Clinical Medicine, University of Copenhagen, Copenhagen, Denmark*
10. *Department of Clinical Immunology, Aarhus University Hospital, Skejby, Denmark*

Short title: Blood group prediction method

*Corresponding author:
Kati Hyvärinen, PhD, Associate professor
Finnish Red Cross Blood Service
Research and Development
Biomedicum Helsinki 1, Haartmaninkatu 8, 00290 Helsinki, FINLAND
Phone +358 40 920 3083
kati.hyvarinen@bloodservice.fi

Table A. Accuracy metrics for the Finnish random forest models in the Finnish test data set. 3
Table B. Accuracy metrics for the Finnish random forest models in the Finnish train data set. 4
Table C. Accuracy metrics for the Finnish random forest models in the Finnish full data set. 5
Table D. Accuracy metrics for the Finnish gradient boosting models in the Finnish test data set. 6
Table E. Accuracy metrics for the Finnish random forest models in the Danish full data set. 7
Table F. Accuracy metrics for the Danish random forest models in the Danish test data set. 8
Table G. Accuracy metrics for the Danish random forest models in the Danish train data set. 9
Table H. Accuracy metrics for the Danish random forest models in the Danish full data set. 10
Table I. Characteristics of the Danish random forest classification models. 11
Table J. Blood group/HPA-1 genes and genetic regions 12
Fig A. Confusion matrices for the Finnish random forest models in the Finnish test data set. 13
Fig B. Confusion matrices for the Finnish random forest models in the Finnish train data set. 14
Fig C. Confusion matrices for the Finnish random forest models in the Finnish full data set 15
Fig D. Receiver operating characteristic and precision-recall curves for the Finnish random forest models in the Finnish test data set 16
Fig E. Posterior probability boxplots for the Finnish random forest models in the Finnish test data set 17
Fig F. Posterior probability boxplots for the Finnish random forest models in the Finnish train data set 18
Fig G. Posterior probability boxplots for the Finnish random forest models in the Finnish full data set. 19
Fig H. Confusion matrices for the Finnish gradient boosting models in the Finnish test data set 20
Fig I. Posterior probability boxplots for the Finnish gradient boosting models in the Finnish test data set 21
Fig J. Confusion matrices for the Danish random forest models in the Danish train data set 22
Fig K. Confusion matrices for the Danish random forest models in the Danish test data set 23
Fig L. Confusion matrices for the Danish random forest models in the Danish full data set 24

Table A. Accuracy metrics for the Finnish random forest models in the Finnish test data set.

Blood group/HPA system	Antigen[a]	Sensitivity	Specificity	Positive predictive value	Negative predictive value	Balanced accuracy
ABO	A	0.997	1.000	1.000	0.996	0.999
ABO	A_1	1.000	1.000	1.000	1.000	1.000
ABO	A_2	1.000	1.000	1.000	1.000	1.000
ABO	AB	1.000	1.000	1.000	1.000	1.000
ABO	B	1.000	1.000	1.000	1.000	1.000
ABO	O	1.000	0.996	0.997	1.000	0.998
Cartwright	Yt^b	1.000	1.000	1.000	1.000	1.000
Colton	Co^a	1.000	1.000	1.000	1.000	1.000
Colton	Co^b	1.000	0.952	0.996	1.000	0.976
Dombrock	Do^a	1.000	1.000	1.000	1.000	1.000
Dombrock	Dob	1.000	1.000	1.000	1.000	1.000
Duffy	Fy^a	1.000	1.000	1.000	1.000	1.000
Duffy	Fy^b	0.979	1.000	1.000	0.993	0.990
Gerbich	Ls^a	1.000	0.667	0.989	1.000	0.833
HPA-1	HPA-1a	0.900	1.000	1.000	0.991	0.950
HPA-1	HPA-1b	1.000	1.000	1.000	1.000	1.000
Kell	K	1.000	1.000	1.000	1.000	1.000
Kell	Kp^a	1.000	1.000	1.000	1.000	1.000
Kell	Ul^a	1.000	1.000	1.000	1.000	1.000
Kidd	Jk^a	1.000	1.000	1.000	1.000	1.000
Kidd	Jk^b	0.995	1.000	1.000	0.998	0.997
Landsteiner-Wiener	LW^b	0.994	1.000	1.000	0.889	0.997
Lewis	Le^a	0.991	1.000	1.000	0.929	0.996
Lewis	Le^b	0.880	1.000	1.000	0.971	0.940
Lutheran	Lu^a	1.000	1.000	1.000	1.000	1.000
MNS	M	0.972	0.994	0.959	0.996	0.983
MNS	N	0.992	0.986	0.980	0.994	0.989
MNS	S	1.000	1.000	1.000	1.000	1.000
MNS	s	1.000	1.000	1.000	1.000	1.000
P1PK	P1	1.000	0.979	0.938	1.000	0.990
Rh	C	0.978	1.000	1.000	0.982	0.989
Rh	c	1.000	0.994	0.976	1.000	0.997
Rh	C^W	0.996	1.000	1.000	0.905	0.998
Rh	C^x	0.986	1.000	1.000	0.909	0.993
Rh	D	0.970	0.993	0.982	0.988	0.982
Rh	E	1.000	0.975	0.994	1.000	0.987
Rh	e	1.000	0.998	0.957	1.000	0.999
Rh	hr^B	1.000	0.998	0.955	1.000	0.999
Rh	hr^S	0.933	0.986	0.636	0.998	0.960

[a] O, A_1, and A_2 in this column refer to phenotype.

Table B. Accuracy metrics for the Finnish random forest models in the Finnish train data set.

Blood group/HPA system	Antigen[a]	Sensitivity	Specificity	Positive predictive value	Negative predictive value	Balanced accuracy
ABO	A	1.000	1.000	1.000	1.000	1.000
ABO	A_1	1.000	1.000	1.000	1.000	1.000
ABO	A_2	1.000	1.000	1.000	1.000	1.000
ABO	AB	1.000	1.000	1.000	1.000	1.000
ABO	B	1.000	0.955	0.929	1.000	0.977
ABO	O	1.000	0.929	0.957	1.000	0.964
Cartwright	Yt^b	1.000	1.000	1.000	1.000	1.000
Colton	Co^a	0.000	1.000	NA	0.997	0.500
Colton	Co^b	1.000	1.000	1.000	1.000	1.000
Dombrock	Do^a	1.000	1.000	1.000	1.000	1.000
Dombrock	Do^b	1.000	1.000	1.000	1.000	1.000
Duffy	Fy^a	1.000	1.000	1.000	1.000	1.000
Duffy	Fy^b	0.986	0.998	0.993	0.995	0.992
Gerbich	Ls^a	1.000	0.667	0.989	1.000	0.833
HPA-1	HPA-1a	1.000	1.000	1.000	1.000	1.000
HPA-1	HPA-1b	0.944	1.000	1.000	0.933	0.972
Kell	K	1.000	1.000	1.000	1.000	1.000
Kell	Kp^a	1.000	1.000	1.000	1.000	1.000
Kell	Ul^a	1.000	1.000	1.000	1.000	1.000
Kidd	Jk^a	1.000	1.000	1.000	1.000	1.000
Kidd	Jk^b	1.000	1.000	1.000	1.000	1.000
Landsteiner-Wiener	LW^b	1.000	1.000	1.000	1.000	1.000
Lewis	Le^a	1.000	1.000	1.000	1.000	1.000
Lewis	Le^b	0.920	0.990	0.958	0.981	0.955
Lutheran	Lu^a	1.000	0.952	0.998	1.000	0.976
MNS	M	0.972	0.998	0.986	0.996	0.985
MNS	N	0.983	0.994	0.992	0.989	0.989
MNS	S	1.000	1.000	1.000	1.000	1.000
MNS	s	1.000	1.000	1.000	1.000	1.000
P1PK	P1	0.968	0.990	0.968	0.990	0.979
Rh	C	0.992	0.998	0.992	0.998	0.995
Rh	c	0.982	0.994	0.993	0.985	0.988
Rh	C^W	0.982	0.998	0.994	0.993	0.990
Rh	C^x	1.000	1.000	1.000	1.000	1.000
Rh	D	0.957	1.000	1.000	0.998	0.978
Rh	E	1.000	1.000	1.000	1.000	1.000
Rh	e	0.998	1.000	1.000	0.950	0.999
Rh	hr^B	1.000	0.993	0.800	1.000	0.996
Rh	hr^S	0.952	1.000	1.000	0.998	0.976

[a] O, A_1, and A_2 in this column refer to phenotype.

Table C. Accuracy metrics for the Finnish random forest models in the Finnish full data set.

Blood group/HPA system	Antigen[a]	Sensitivity	Specificity	Positive predictive value	Negative predictive value	Balanced accuracy
ABO	A	0.999	1.000	1.000	0.998	0.999
ABO	A_1	1.000	1.000	1.000	1.000	1.000
ABO	A_2	1.000	1.000	1.000	1.000	1.000
ABO	AB	1.000	0.998	0.999	1.000	0.999
ABO	B	1.000	1.000	1.000	1.000	1.000
ABO	O	1.000	0.963	0.977	1.000	0.981
Cartwright	Yt^b	1.000	1.000	1.000	1.000	1.000
Colton	Co^a	1.000	1.000	1.000	1.000	1.000
Colton	Co^b	1.000	0.976	0.998	1.000	0.988
Dombrock	Do^a	1.000	1.000	1.000	1.000	1.000
Dombrock	Do^b	1.000	1.000	1.000	1.000	1.000
Duffy	Fy^a	1.000	1.000	1.000	1.000	1.000
Duffy	Fy^b	0.986	0.999	0.997	0.995	0.993
Gerbich	Ls^a	1.000	0.833	0.995	1.000	0.917
HPA-1	HPA-1a	1.000	1.000	1.000	1.000	1.000
HPA-1	HPA-1b	0.971	1.000	1.000	0.964	0.986
Kell	K	1.000	1.000	1.000	1.000	1.000
Kell	Kp^a	1.000	1.000	1.000	1.000	1.000
Kell	Ul^a	1.000	1.000	1.000	1.000	1.000
Kidd	Jk^a	1.000	1.000	1.000	1.000	1.000
Kidd	Jk^b	0.997	1.000	1.000	0.999	0.999
Landsteiner-Wiener	LW^b	0.997	1.000	1.000	0.944	0.998
Lewis	Le^a	0.996	1.000	1.000	0.963	0.998
Lewis	Le^b	0.940	0.995	0.979	0.985	0.968
Lutheran	Lu^a	1.000	0.976	0.999	1.000	0.988
MNS	M	0.972	0.996	0.972	0.996	0.984
MNS	N	0.988	0.988	0.983	0.991	0.988
MNS	S	1.000	1.000	1.000	1.000	1.000
MNS	s	1.000	1.000	1.000	1.000	1.000
P1PK	P1	0.984	0.984	0.952	0.995	0.984
Rh	C	0.996	0.998	0.992	0.999	0.997
Rh	c	0.983	0.995	0.994	0.986	0.989
Rh	C^W	0.976	0.999	0.997	0.991	0.988
Rh	C^x	1.000	0.996	0.999	1.000	0.998
Rh	D	0.978	0.999	0.978	0.999	0.988
Rh	E	0.993	1.000	1.000	0.953	0.997
Rh	e	0.997	1.000	1.000	0.927	0.999
Rh	hr^B	0.871	0.995	0.818	0.996	0.933
Rh	hr^S	0.976	0.999	0.976	0.999	0.988

[a] O, A_1, and A_2 in this column refer to phenotype.

Table D. Accuracy metrics for the Finnish gradient boosting models in the Finnish test data set.

Blood group/HPA system	Antigen[a]	Sensitivity	Specificity	Positive predictive value	Negative predictive value	Balanced accuracy
ABO	A	0.997	0.984	0.989	0.996	0.990
ABO	A_1	1.000	0.955	0.929	1.000	0.977
ABO	A_2	0.905	0.846	0.905	0.846	0.875
ABO	AB	1.000	0.915	0.993	1.000	0.957
ABO	B	1.000	0.939	0.995	1.000	0.969
ABO	O	1.000	0.973	0.980	1.000	0.986
Cartwright	Yt^b	1.000	1.000	1.000	1.000	1.000
Colton	Co^a	0.000	1.000	NA	0.997	0.500
Colton	Co^b	1.000	0.976	0.998	1.000	0.988
Dombrock	Do^a	1.000	0.997	0.996	1.000	0.998
Dombrock	Do^b	1.000	1.000	1.000	1.000	1.000
Duffy	Fy^a	1.000	0.990	0.980	1.000	0.995
Duffy	Fy^b	0.959	1.000	1.000	0.987	0.979
Gerbich	Ls^a	1.000	0.000	0.968	NA	0.500
HPA-1	HPA-1a	0.800	1.000	1.000	0.981	0.900
HPA-1	HPA-1b	0.941	0.923	0.941	0.923	0.932
Kell	K	1.000	0.929	0.996	1.000	0.964
Kell	Kp^a	1.000	1.000	1.000	1.000	1.000
Kell	Ul^a	1.000	0.882	0.979	1.000	0.941
Kidd	Jk^a	0.988	1.000	1.000	0.995	0.994
Kidd	Jk^b	1.000	0.988	0.974	1.000	0.994
Landsteiner-Wiener	LW^b	0.994	1.000	1.000	0.889	0.997
Lewis	Le^a	1.000	0.923	0.991	1.000	0.962
Lewis	Le^b	0.880	0.960	0.846	0.970	0.920
Lutheran	Lu^a	1.000	1.000	1.000	1.000	1.000
MNS	M	0.972	1.000	1.000	0.996	0.986
MNS	N	0.971	0.991	0.987	0.980	0.981
MNS	S	1.000	0.984	0.982	1.000	0.992
MNS	s	1.000	0.998	0.986	1.000	0.999
P1PK	P1	0.900	0.990	0.964	0.969	0.945
Rh	C	0.971	0.985	0.981	0.976	0.978
Rh	c	0.943	0.973	0.898	0.985	0.958
Rh	C^W	1.000	1.000	1.000	1.000	1.000
Rh	C^x	0.993	0.900	0.987	0.947	0.947
Rh	D	0.959	0.967	0.920	0.983	0.963
Rh	E	1.000	0.958	0.990	1.000	0.979
Rh	e	0.864	0.998	0.950	0.995	0.931
Rh	hr^B	0.952	0.998	0.952	0.998	0.975
Rh	hr^S	1.000	0.989	0.714	1.000	0.995

[a] O, A_1, and A_2 in this column refer to phenotype. NA, not available.

Table E. Accuracy metrics for the Finnish random forest models in the Danish full data set.

Blood group/HPA system	Antigen[a]	Sensitivity	Specificity	Positive predictive value	Negative predictive value	Balanced accuracy
ABO	A	0.993	0.998	0.998	0.991	0.995
ABO	A_1	0.972	0.988	0.963	0.991	0.980
ABO	A_2	0.984	0.803	0.931	0.949	0.894
ABO	AB	0.999	0.997	1.000	0.985	0.998
ABO	B	1.000	0.975	0.997	0.998	0.987
ABO	O	0.998	0.992	0.993	0.998	0.995
Cartwright	Yt^b	0.999	0.996	1.000	0.991	0.998
Colton	Co^a	0.667	1.000	0.800	0.999	0.833
Colton	Co^b	0.999	0.854	0.985	0.989	0.926
Dombrock	Do^a	0.999	0.999	0.999	0.999	0.999
Dombrock	Do^b	0.997	1.000	0.999	1.000	0.999
Duffy	Fy^a	0.963	0.996	0.992	0.982	0.979
Duffy	Fy^b	0.945	0.998	0.990	0.987	0.971
HPA-1	HPA-1a	1.000	1.000	1.000	1.000	1.000
HPA-1	HPA-1b	0.997	0.881	0.950	0.993	0.939
Kell	K	0.998	0.834	0.986	0.979	0.916
Kell	Kp^a	1.000	0.870	0.997	0.981	0.935
Kidd	Jk^a	0.991	0.998	0.995	0.997	0.995
Kidd	Jk^b	0.999	0.325	0.357	0.999	0.662
Landsteiner-Wiener	Lw^b	0.000	1.000	NA	0.010	0.500
Lewis	Le^a	0.981	0.982	0.996	0.914	0.981
Lewis	Le^b	0.465	0.994	0.983	0.707	0.729
Lutheran	Lu^a	0.998	0.914	0.992	0.975	0.956
MNS	M	0.942	0.999	0.996	0.984	0.970
MNS	N	0.966	0.928	0.842	0.986	0.947
MNS	s	0.976	1.000	0.996	0.998	0.988
MNS	S	0.993	0.998	0.998	0.993	0.995
P1PK	P1	0.000	1.000	NA	0.774	0.500
Rh	c	0.000	1.000	NA	0.822	0.500
Rh	C	0.999	0.293	0.450	0.997	0.646
Rh	C^W	1.000	0.835	0.994	0.987	0.918
Rh	D	0.977	0.591	0.382	0.990	0.784
Rh	e	0.964	1.000	0.992	0.999	0.982
Rh	E	0.999	0.985	0.994	0.998	0.992

[a] O, A_1, and A_2 in this column refer to phenotype.

Table F. Accuracy metrics for the Danish random forest models in the Danish test data set.

Blood group/HPA system	Antigen[a]	Sensitivity	Specificity	Positive predictive value	Negative predictive value	Balanced accuracy
ABO	A	0.999	0.998	0.998	0.999	0.998
ABO	A_1	0.971	0.992	0.974	0.991	0.981
ABO	A_2	0.975	0.810	0.933	0.922	0.893
ABO	AB	1.000	0.996	1.000	0.997	0.998
ABO	B	1.000	0.998	1.000	0.998	0.999
ABO	O	0.999	0.997	0.998	0.999	0.998
Cartwright	Yt^a	1.000	1.000	0.933	1.000	1.000
Cartwright	Yt^b	0.999	1.000	1.000	0.985	0.999
Colton	Co^a	0.500	1.000	0.750	0.999	0.750
Colton	Co^b	0.993	0.950	0.995	0.928	0.971
Dombrock	Do^a	1.000	0.999	0.999	1.000	1.000
Dombrock	Do^b	0.996	1.000	0.998	0.999	0.998
Duffy	Fy^a	0.995	0.998	0.996	0.998	0.997
Duffy	Fy^b	0.963	0.999	0.995	0.991	0.981
HPA-1	HPA-1a	1.000	1.000	1.000	1.000	1.000
HPA-1	HPA-1b	0.994	1.000	1.000	0.988	0.997
Kell	K	0.996	0.906	0.992	0.956	0.951
Kell	k	0.681	1.000	0.942	0.997	0.840
Kell	Kp^a	0.999	0.888	0.997	0.956	0.944
Kell	Kp^b	0.400	1.000	1.000	0.999	0.700
Kidd	Jk^a	0.996	0.998	0.995	0.999	0.997
Kidd	Jk^b	0.996	0.999	0.997	0.998	0.997
Knops	Kn^a	0.000	1.000	NA	0.999	0.500
Knops	Kn^b	1.000	1.000	1.000	1.000	1.000
Lewis	Le^a	0.996	0.986	0.997	0.981	0.991
Lewis	Le^b	0.499	0.979	0.948	0.717	0.739
Lutheran	Lu^a	0.997	0.931	0.994	0.971	0.964
Lutheran	Lu^b	0.750	1.000	0.923	0.999	0.875
MNS	M	0.989	0.998	0.993	0.997	0.994
MNS	N	0.981	0.990	0.975	0.993	0.986
MNS	S	0.997	0.997	0.997	0.997	0.997
MNS	s	0.985	0.999	0.995	0.998	0.992
P1PK	P1	0.939	0.996	0.986	0.982	0.968
Rh	C	0.994	0.999	0.999	0.997	0.997
Rh	c	0.997	0.999	0.995	0.999	0.998
Rh	C^W	0.998	0.898	0.997	0.952	0.948
Rh	D	0.994	0.999	0.997	0.998	0.997
Rh	E	0.999	0.989	0.996	0.998	0.994
Rh	e	0.962	1.000	0.993	0.999	0.981
Vel	Vel	0.568	1.000	0.955	0.999	0.784

[a] O, A_1, and A_2 in this column refer to phenotype.

Table G. Accuracy metrics for the Danish random forest models in the Danish train data set.

Blood group/HPA system	Antigen[a]	Sensitivity	Specificity	Positive predictive value	Negative predictive value	Balanced accuracy
ABO	A	0.999	0.998	0.998	0.998	0.998
ABO	AB	1.000	0.997	1.000	0.996	0.998
ABO	B	1.000	0.997	1.000	0.998	0.999
ABO	O	0.999	0.997	0.998	0.999	0.998
ABO	A_1	0.976	0.991	0.972	0.992	0.983
ABO	A_2	0.987	0.797	0.929	0.959	0.892
Cartwright	Yt^a	1.000	1.000	1.000	1.000	1.000
Cartwright	Yt^b	1.000	0.993	0.999	0.996	0.996
Colton	Co^a	0.500	0.999	0.600	0.999	0.750
Colton	Co^b	0.994	0.934	0.993	0.942	0.964
Dombrock	Do^a	0.999	0.999	0.998	0.999	0.999
Dombrock	Do^b	0.998	1.000	1.000	1.000	0.999
Duffy	Fy^a	0.995	0.998	0.995	0.998	0.997
Duffy	Fy^b	0.959	0.999	0.995	0.990	0.979
HPA-1	HPA-1a	1.000	1.000	1.000	1.000	1.000
HPA-1	HPA-1b	1.000	1.000	1.000	1.000	1.000
Kell	K	0.996	0.911	0.992	0.953	0.954
Kell	k	0.562	1.000	0.932	0.996	0.781
Kell	Kp^a	0.999	0.929	0.998	0.951	0.964
Kell	Kp^b	0.600	1.000	1.000	1.000	0.800
Kidd	Jk^a	0.993	0.998	0.995	0.998	0.996
Kidd	Jk^b	0.995	0.999	0.997	0.998	0.997
Knops	Kn^a	1.000	1.000	1.000	1.000	1.000
Knops	Kn^b	1.000	1.000	1.000	1.000	1.000
Lewis	Le^a	0.995	0.978	0.995	0.978	0.987
Lewis	Le^b	0.487	0.974	0.936	0.712	0.731
Lutheran	Lu^a	0.996	0.933	0.994	0.957	0.965
Lutheran	Lu^b	0.563	1.000	1.000	0.998	0.781
MNS	M	0.988	0.998	0.993	0.997	0.993
MNS	N	0.983	0.990	0.976	0.993	0.987
MNS	S	0.997	0.998	0.998	0.997	0.998
MNS	s	0.988	1.000	0.995	0.999	0.994
P1PK	P1	0.928	0.994	0.980	0.979	0.961
Rh	C	0.994	0.999	0.998	0.997	0.997
Rh	c	0.995	0.999	0.995	0.999	0.997
Rh	D	0.994	0.999	0.997	0.998	0.996
Rh	E	0.999	0.991	0.996	0.997	0.995
Rh	e	0.972	1.000	0.995	0.999	0.986
Rh	C^W	0.999	0.907	0.997	0.959	0.953
Vel	Vel	0.676	1.000	1.000	0.999	0.838

[a] O, A_1, and A_2 in this column refer to phenotype.

Table H. Accuracy metrics for the Danish random forest models in the Danish full data set.

Blood group/HPA system	Antigen[a]	Sensitivity	Specificity	Positive predictive value	Negative predictive value	Balanced accuracy
ABO	A	0.999	0.998	0.999	0.999	0.999
ABO	AB	1.000	0.998	1.000	0.997	0.999
ABO	B	1.000	0.997	1.000	0.998	0.999
ABO	O	0.999	0.998	0.998	0.999	0.998
ABO	A_1	0.977	0.991	0.974	0.992	0.984
ABO	A_2	0.987	0.795	0.929	0.959	0.891
Cartwright	Yt^a	1.000	1.000	0.966	1.000	1.000
Cartwright	Yt^b	1.000	0.996	1.000	0.996	0.998
Colton	Co^a	0.667	1.000	0.800	0.999	0.833
Colton	Co^b	0.995	0.930	0.993	0.954	0.963
Dombrock	Do^a	0.999	0.999	0.999	1.000	0.999
Dombrock	Do^b	0.998	1.000	0.999	1.000	0.999
Duffy	Fy^a	0.996	0.998	0.996	0.998	0.997
Duffy	Fy^b	0.961	0.999	0.996	0.991	0.980
HPA-1	HPA-1a	1.000	1.000	1.000	1.000	1.000
HPA-1	HPA-1b	0.997	1.000	1.000	0.994	0.999
Kell	K	0.996	0.915	0.993	0.953	0.956
Kell	k	0.676	0.999	0.925	0.997	0.838
Kell	Kp^a	0.999	0.924	0.998	0.956	0.961
Kell	Kp^b	0.500	1.000	1.000	1.000	0.750
Kidd	Jk^a	0.995	0.998	0.995	0.998	0.997
Kidd	Jk^b	0.995	0.999	0.997	0.998	0.997
Knops	Kn^a	0.500	1.000	1.000	0.999	0.750
Knops	Kn^b	1.000	1.000	1.000	1.000	1.000
Lewis	Le^a	0.996	0.982	0.996	0.981	0.989
Lewis	Le^b	0.490	0.982	0.954	0.714	0.736
Lutheran	Lu^a	0.997	0.932	0.994	0.964	0.964
Lutheran	Lu^b	0.656	1.000	0.955	0.999	0.828
MNS	M	0.989	0.998	0.994	0.997	0.993
MNS	N	0.982	0.991	0.976	0.993	0.987
MNS	S	0.997	0.997	0.997	0.997	0.997
MNS	s	0.986	1.000	0.995	0.999	0.993
P1PK	P1	0.934	0.995	0.983	0.981	0.965
Rh	C	0.995	0.999	0.999	0.997	0.997
Rh	c	0.996	0.999	0.995	0.999	0.997
Rh	D	0.994	0.999	0.997	0.998	0.997
Rh	E	0.999	0.991	0.997	0.997	0.995
Rh	e	0.969	1.000	0.993	0.999	0.984
Rh	C^W	0.999	0.908	0.997	0.965	0.953
Vel	Vel	0.622	1.000	0.920	0.999	0.811

[a] O, A_1, and A_2 in this column refer to phenotype.

Table I. Characteristics of the Danish random forest classification models.

Blood group/HPA system	Antigen[a]	Genes analyzed	n(variants available)	n(model variants)	Prediction error[b]
ABO	ABO	*ABO*	615	603	1.80E-03
ABO	A_1	*ABO*	615	586	1.17E-02
ABO	A_2	*ABO*	615	480	5.95E-02
Cartwright	Yt^a	*ACHE*	76	21	1.47E-04
Cartwright	Yt^b	*ACHE*	76	44	6.15E-04
Colton	Co^a	*AQP1*	69	36	9.20E-04
Colton	Co^b	*AQP1*	69	41	8.33E-03
Dombrock	Do^a	*ART4*	141	104	8.60E-04
Dombrock	Do^b	*ART4*	141	113	3.79E-04
Duffy	Fy^a	*ACKR1*	42	42	2.42E-03
Duffy	Fy^b	*ACKR1*	42	40	7.76E-03
HPA-1	HPA-1a	*ITGB3*	377	20	2.06E-04
HPA-1	HPA-1b	*ITGB3*	377	91	2.28E-03
Kell	K	*KEL*	87	86	8.50E-03
Kell	Kp^a	*KEL*	87	50	2.75E-03
Kidd	Jk^a	*SLC14A1*	509	504	2.47E-03
Kidd	Jk^b	*SLC14A1*	509	492	2.09E-03
Knops	Kn^a	*CR1*	622	110	4.28E-04
Knops	Kn^b	*CR1*	622	31	2.16E-05
Lewis	Le^a	*FUT2. FUT3*	210	206	6.48E-03
Lewis	Le^b	*FUT2. FUT3*	210	203	1.68E-01
Lutheran	Lu^a	*BCAM*	98	95	7.89E-03
Lutheran	Lu^b	*BCAM*	98	77	1.49E-03
MNS	M	*GYPA. GYPB. GYPE*	766	736	3.58E-03
MNS	N	*GYPA. GYPB. GYPE*	766	743	1.15E-02
MNS	S	*GYPA. GYPB. GYPE*	766	702	2.54E-03
MNS	s	*GYPA. GYPB. GYPE*	766	655	1.72E-03
P1PK	P1	*A4GALT. B3GALNT1*	499	489	1.89E-02
Rh	C	*RHCE. RHD*	465	452	2.14E-03
Rh	C^W	*RHCE. RHD*	465	411	3.50E-03
Rh	D	*RHCE. RHD*	465	461	1.66E-03
Rh	E	*RHCE. RHD*	465	464	2.70E-03
Rh	e	*RHCE. RHD*	465	423	9.04E-04
Vel	Vel	*SMIM1*	78	49	9.48E-04

[a] O, A_1, and A_2 in this column refer to phenotype.

[b] Misclassification frequency obtained from out-of-bag data.

Table J. Blood group/HPA-1 genes and genetic regions

Blood group/HPA system	Ensembl gene ID	Genes analyzed	CHR	Start position -2 Kbp GRCh38	End position +2 Kbp GRCh38
ABO	ENSG00000175164	*ABO*	9	133231278	133278024
CartwrightYt	ENSG00000087085	*ACHE*	7	100887994	100898974
Colton	ENSG00000240583	*AQP1*	7	30909853	30927517
Diego	ENSG00000004939	*SLC4A1*	17	44246390	4427014
Dombrock	ENSG00000111339	*ART4*	12	14823569	14845526
Duffy	ENSG00000213088	*ACKR1*	1	159201307	159208500
Gerbich	ENSG00000136732	*GYPC*	2	126654133	126698667
HPA-1	ENSG00000259207	*ITGB3*	17	47251827	47315743
Kell	ENSG00000197993	*KEL*	7	142939114	142964363
Knops	ENSG00000203710	*CR1*	1	207494128	207643765
Kidd	ENSG00000141469	*SLC14A1*	18	45685025	45754520
Landsteiner-Wiener	ENSG00000105371	*ICAM4*	19	10284955	10290522
Lewis	ENSG00000176920	*FUT2*	19	48693971	48707951
Lewis	ENSG00000171124	*FUT3*	19	5840888	5853474
Lutheran	ENSG00000187244	*BCAM*	19	44807071	44823421
MNS	ENSG00000170180	*GYPA*	4	144107303	144142751
MNS	ENSG00000250361	*GYPB*	4	143994104	144021380
MNS	ENSG00000197465	*GYPE*	4	143868864	143907563
P1PK	ENSG00000128274	*A4GALT*	22	42690121	42723298
P1PK	ENSG00000169255	*B3GALNT1*	3	161081883	161107411
Rh	ENSG00000188672	*RHCE*	1	25360249	25432192
Rh	ENSG00000187010	*RHD*	1	25270393	25332445
VEL	ENSG00000235169	*SMIM1*	1	3770749	3777982

CHR, chromosome; GRCh38, Genome Reference Consortium Human Build 38; HPA, human platelet antigen

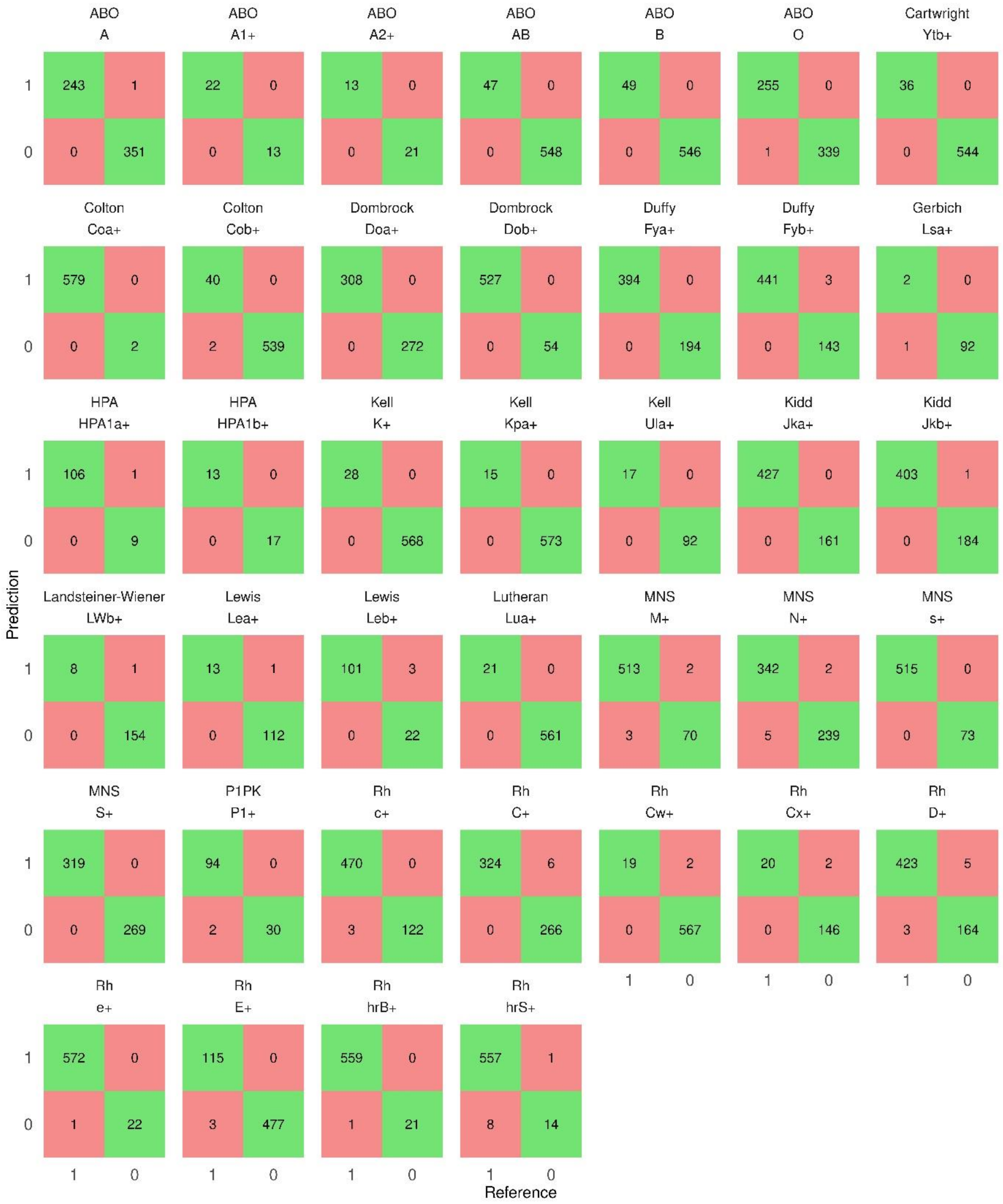

Fig A. Confusion matrices for the Finnish random forest models in the Finnish test data set.

Confusion matrices for the Finnish antigen classification models in the Finnish test data set are presented in alphabetical order of the blood group systems. The RBC antigen/phenotype and HPA-1 typing results are on the x-axis and the model predictions on the y-axis. The antigen-negative samples are denoted by 0 and the antigen-positive samples by 1 on both axes. The numbers of true positive and true negative samples are depicted in the green boxes and the numbers of false positive and false negative samples in the red boxes.

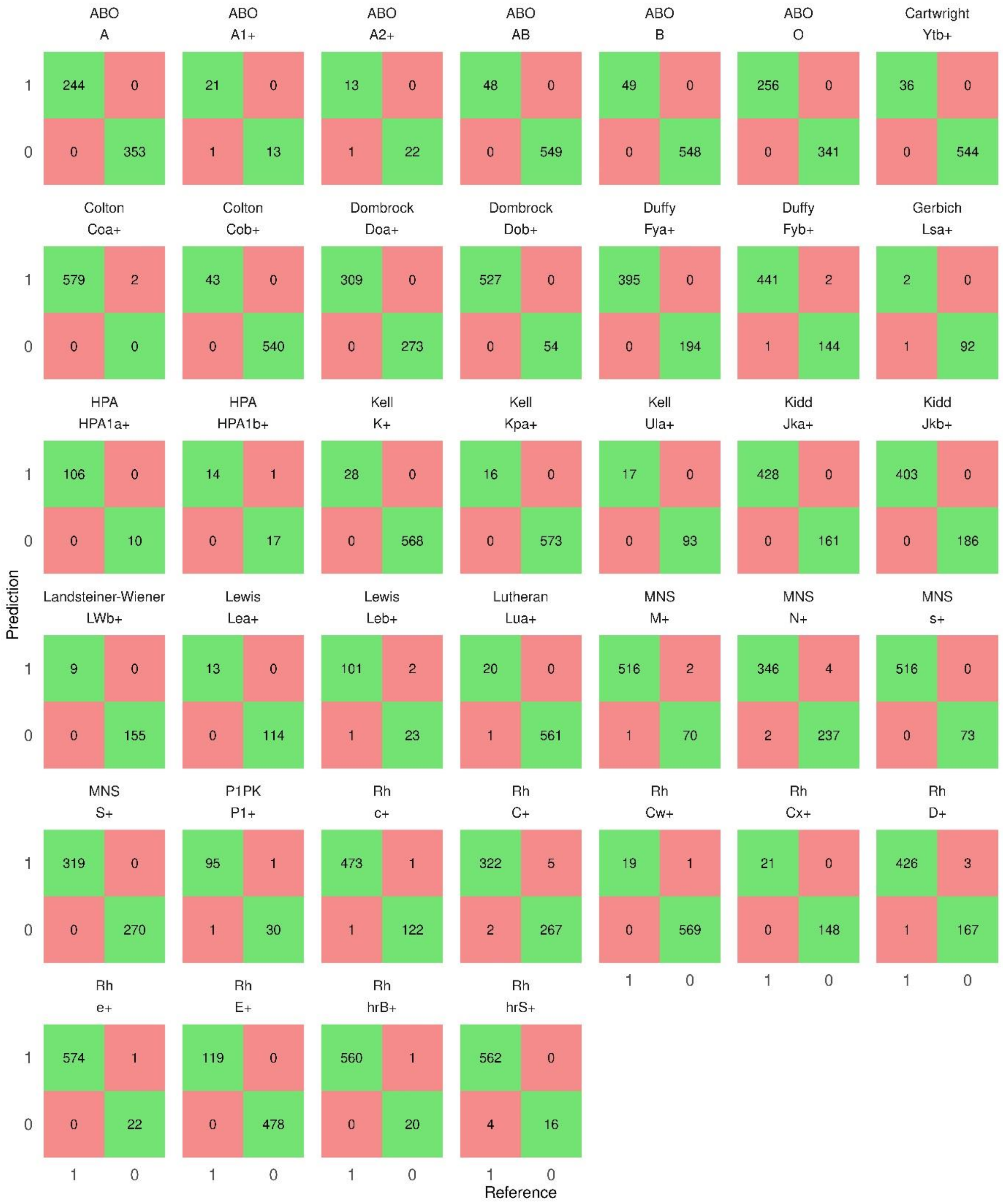

Fig B. Confusion matrices for the Finnish random forest models in the Finnish train data set.

Confusion matrices for the Finnish antigen classification models in the Finnish train data set are presented in alphabetical order of the blood group systems. The RBC antigen/phenotype and HPA-1 typing results are on the x-axis and the model predictions on the y-axis. The antigen-negative samples are denoted by 0 and the antigen-positive samples by 1 on both axes. The numbers of true positive and true negative samples are depicted in the green boxes and the numbers of false positive and false negative samples in the red boxes.

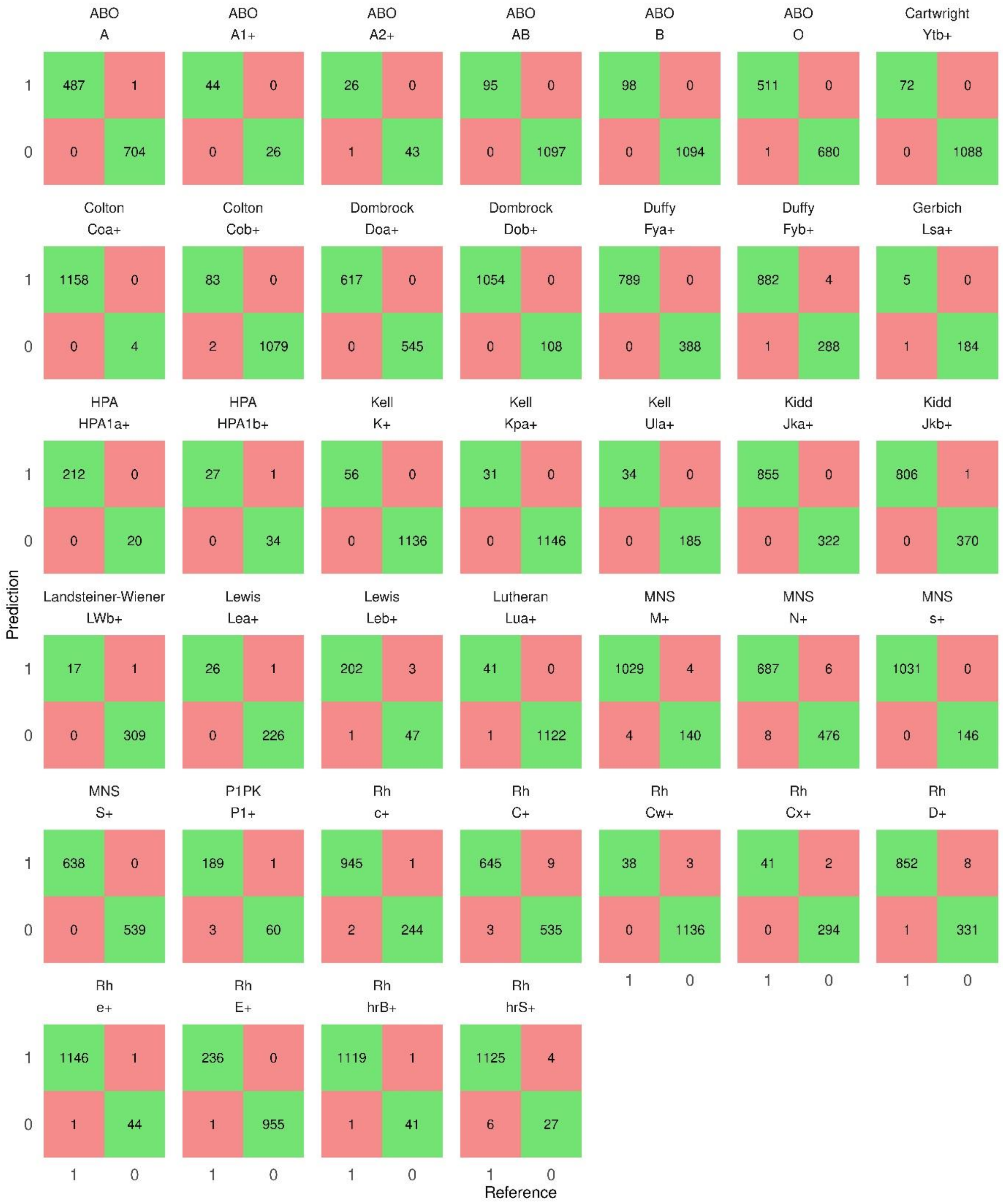

Fig C. Confusion matrices for the Finnish random forest models in the Finnish full data set

Confusion matrices for the Finnish antigen classification models in the Finnish full data set are presented in alphabetical order of the blood group systems. The RBC antigen/phenotype and HPA-1 typing results are on the x-axis and the model predictions on the y-axis. The antigen-negative samples are denoted by 0 and the antigen-positive samples by 1 on both axes. The numbers of true positive and true negative samples are depicted in the green boxes and the numbers of false positive and false negative samples in the red boxes.

A

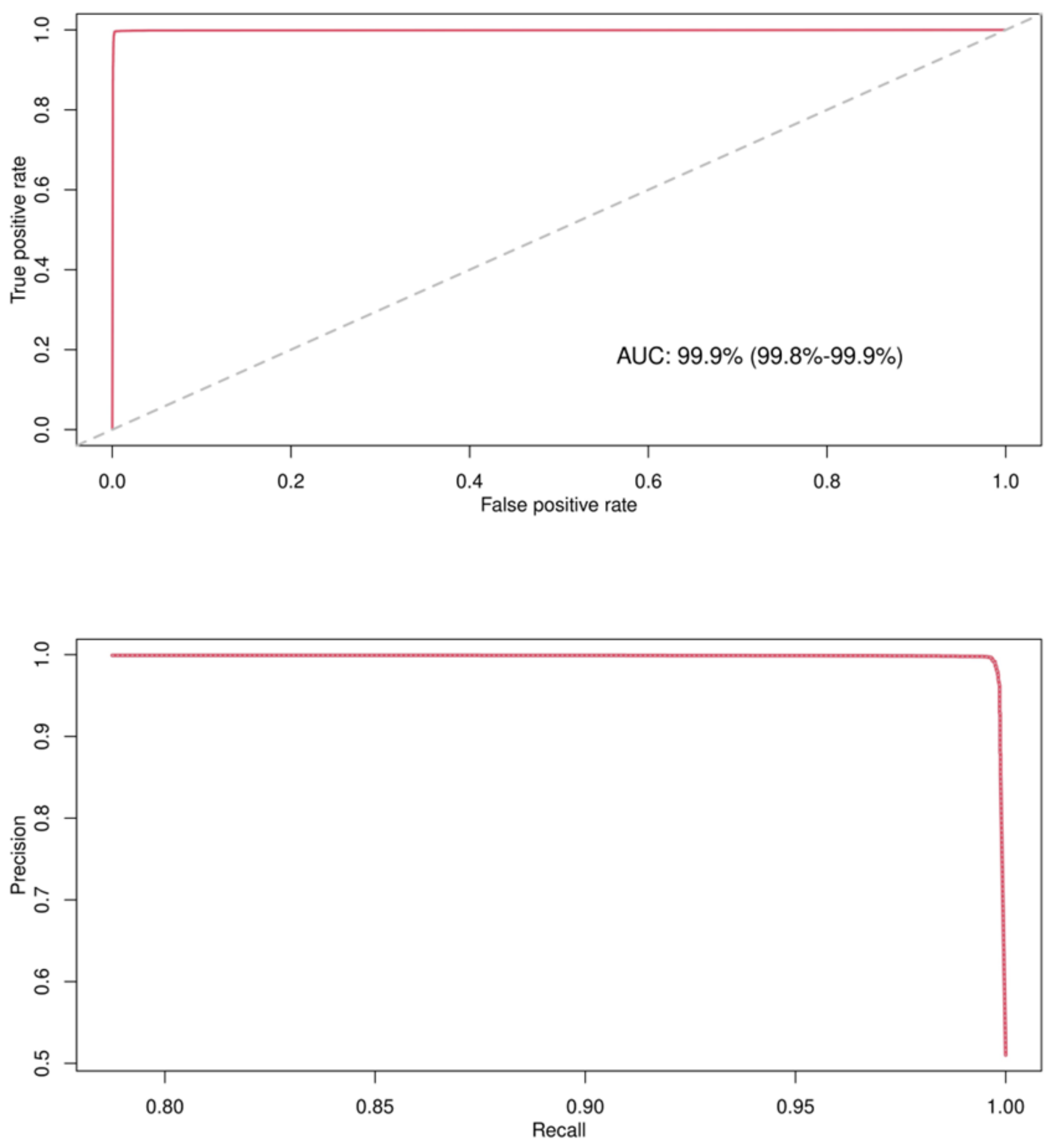

B

Fig D. Receiver operating characteristic and precision-recall curves for the Finnish random forest models in the Finnish test data set

Prediction results from all models are combined together. (A) Receiver operating characteristic curve and area under curve (AUC) for combined test data predictions. (B) Precision-recall curve for combined test data predictions.

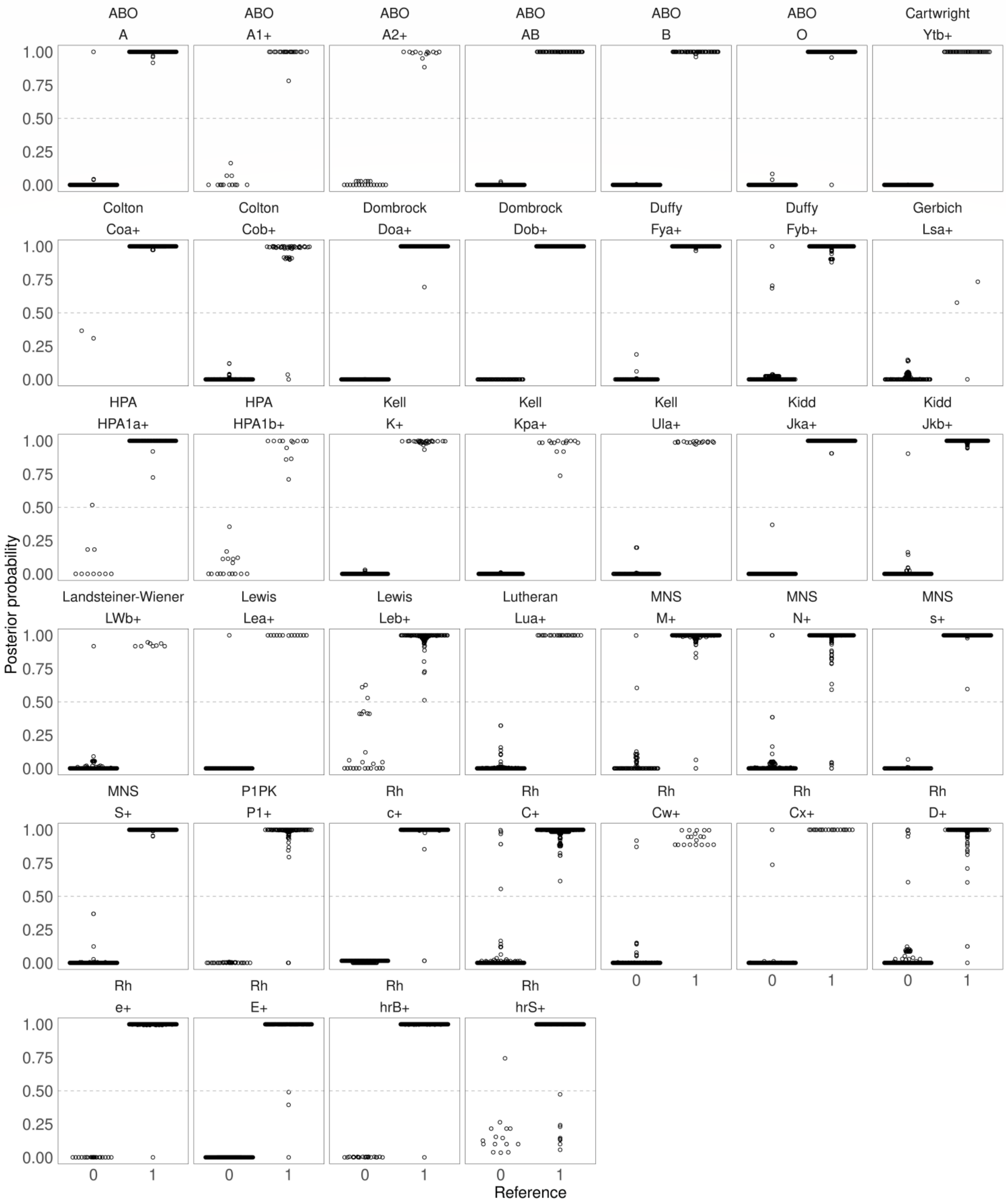

Fig E. Posterior probability boxplots for the Finnish random forest models in the Finnish test data set

Posterior probability boxplots for the Finnish antigen classification models in the Finnish test data set are presented in alphabetical order of the blood group systems. The RBC antigen/phenotype and HPA-1 typing results are on the x-axis and the antigen-negative samples are denoted by 0 and the antigen-positive samples by 1. The posterior probabilities for samples range from 0 to 1 and are presented on the y-axis. Samples are depicted as open circles.

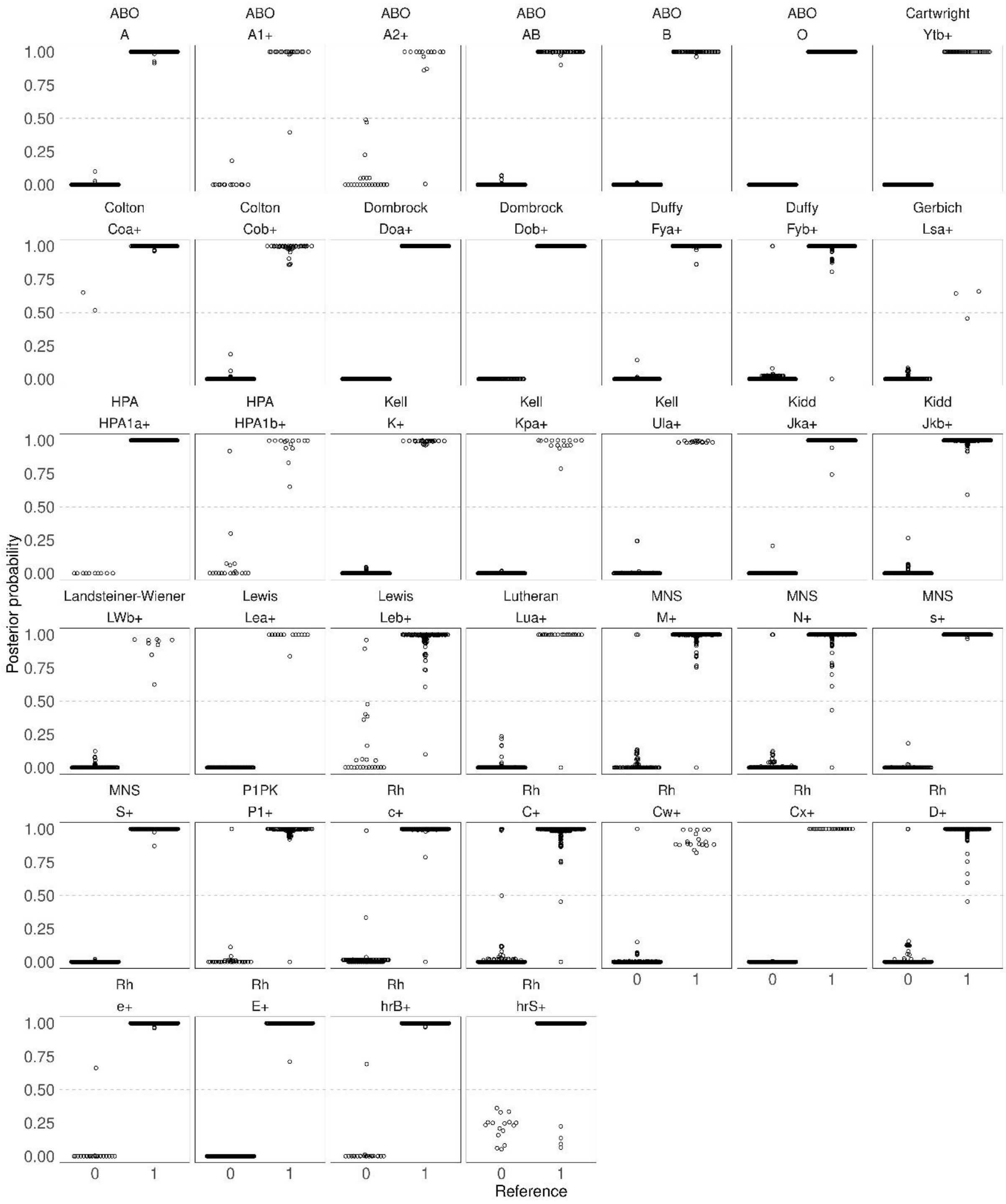

Fig F. Posterior probability boxplots for the Finnish random forest models in the Finnish train data set

Posterior probability boxplots for the Finnish antigen classification models in the Finnish train data set are presented in alphabetical order of the blood group systems. The RBC antigen/phenotype and HPA-1 typing results are on the x-axis and the antigen-negative samples are denoted by 0 and the antigen-positive samples by 1. The posterior probabilities for samples range from 0 to 1 and are presented on the y-axis. Samples are depicted as open circles.

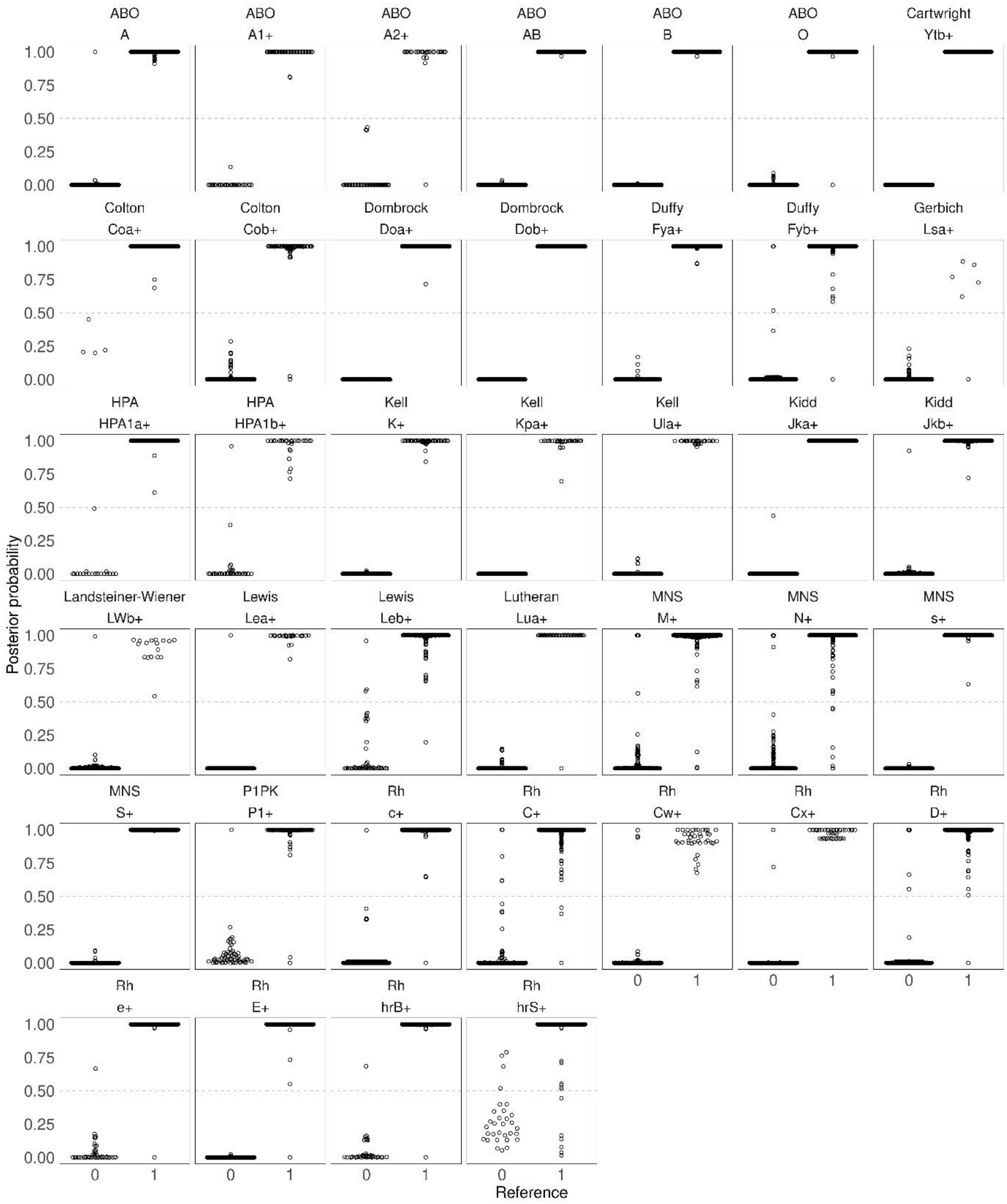

Fig G. Posterior probability boxplots for the Finnish random forest models in the Finnish full data set.

Posterior probability boxplots for the Finnish antigen classification models in the Finnish full data set are presented in alphabetical order of the blood group systems. The RBC antigen/phenotype and HPA-1 typing results are on the x-axis and the antigen-negative samples are denoted by 0 and the antigen-positive samples by 1. The posterior probabilities for samples range from 0 to 1 and are presented on the y-axis. Samples are depicted as open circles.

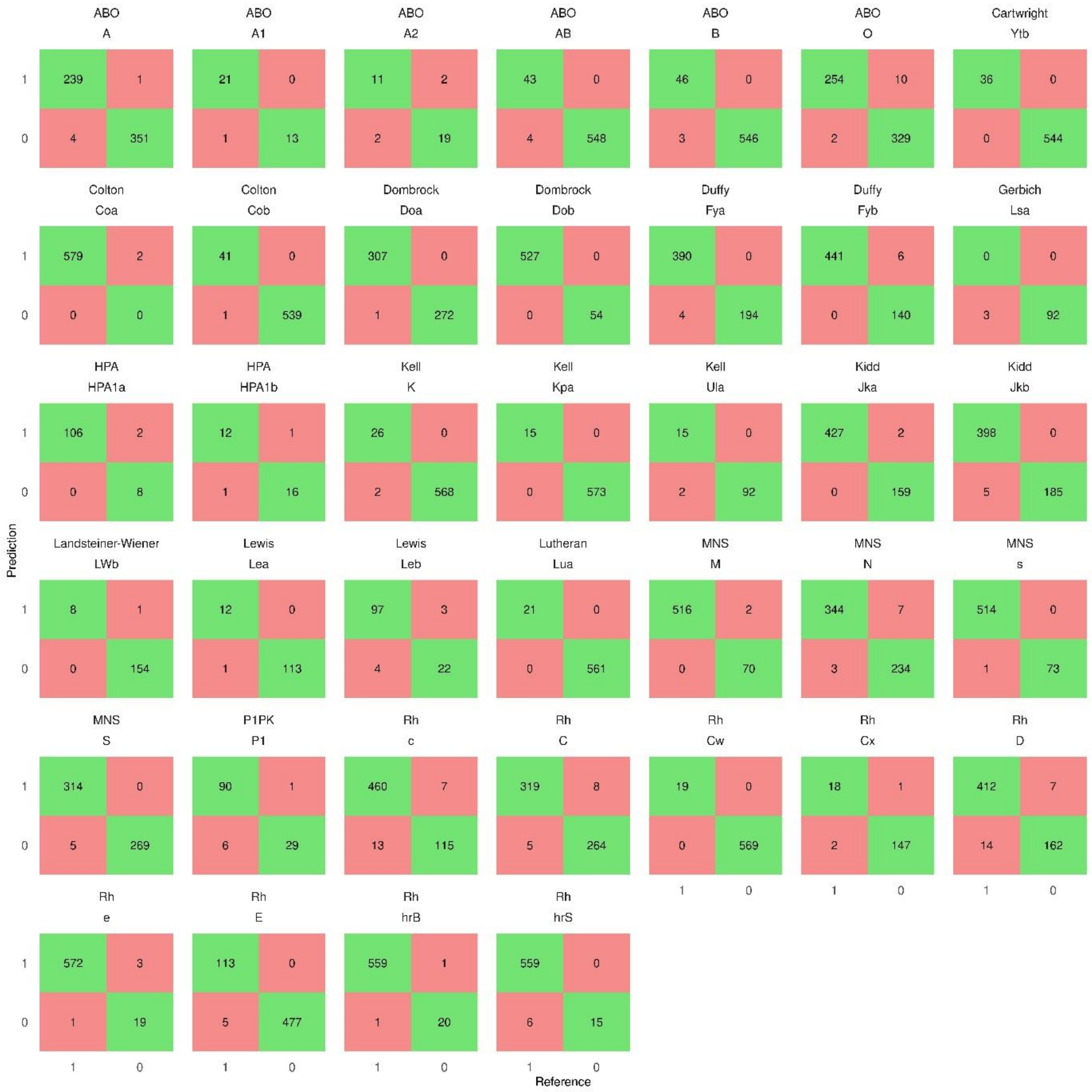

Fig H. Confusion matrices for the Finnish gradient boosting models in the Finnish test data set

Confusion matrices for the Finnish gradient boosting classification models in the Finnish test data set. The RBC antigen/phenotype and HPA-1 typing results are on the x-axis and the model predictions on the y-axis. The antigen-negative samples are denoted by 0 and the antigen-positive samples by 1 on both axes. The numbers of true positive and true negative samples are depicted in the green boxes and the numbers of false positive and false negative samples in the red boxes.

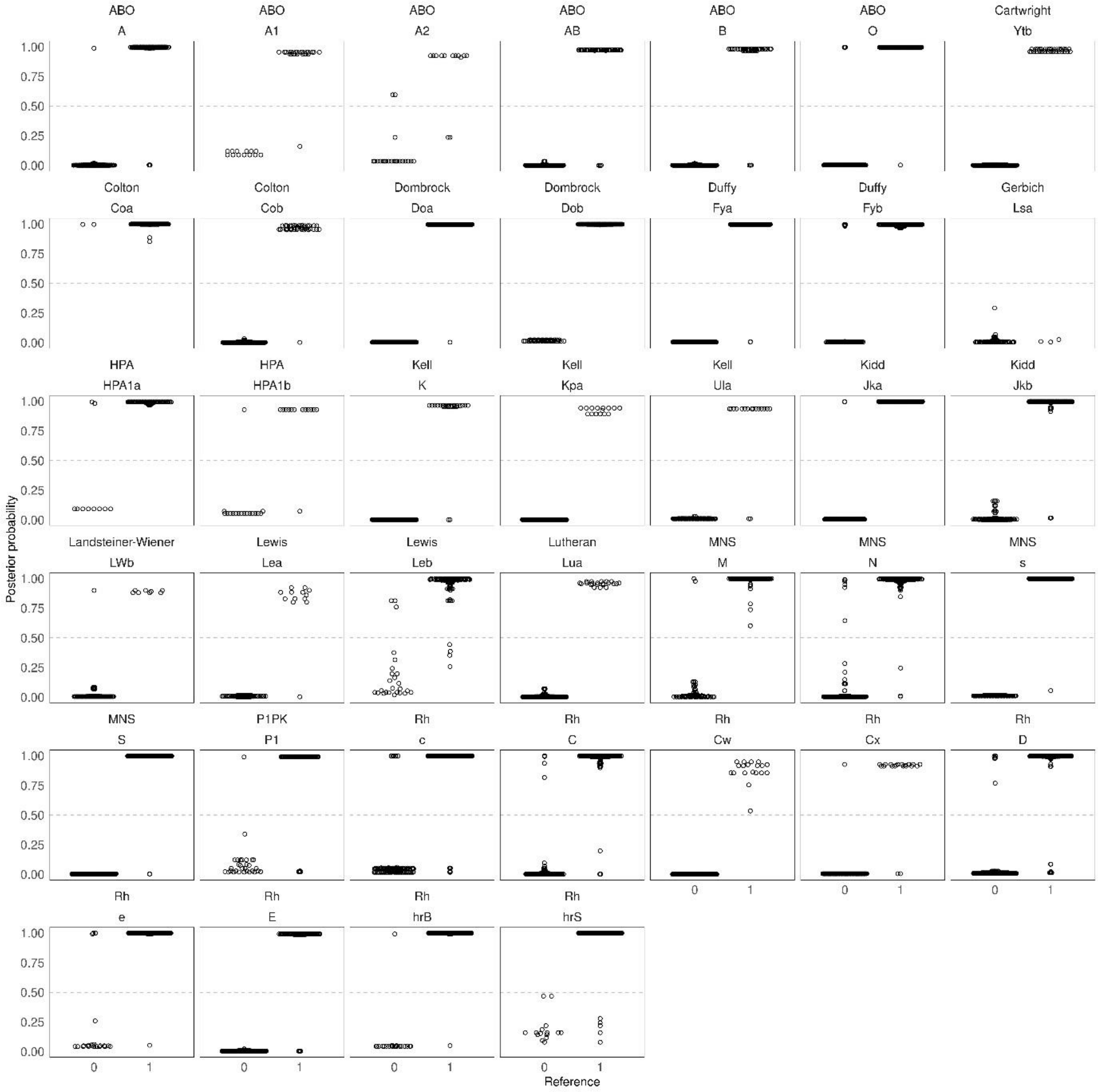

Fig I. Posterior probability boxplots for the Finnish gradient boosting models in the Finnish test data set.

Posterior probability boxplots for the Finnish gradient boosting classification models in the Finnish test data set. The RBC antigen/phenotype and HPA-1 typing results are on the x-axis and the antigen-negative samples are denoted by 0 and the antigen-positive samples by 1. The posterior probabilities for samples range from 0 to 1 and are presented on the y-axis. Samples are depicted as open circles.

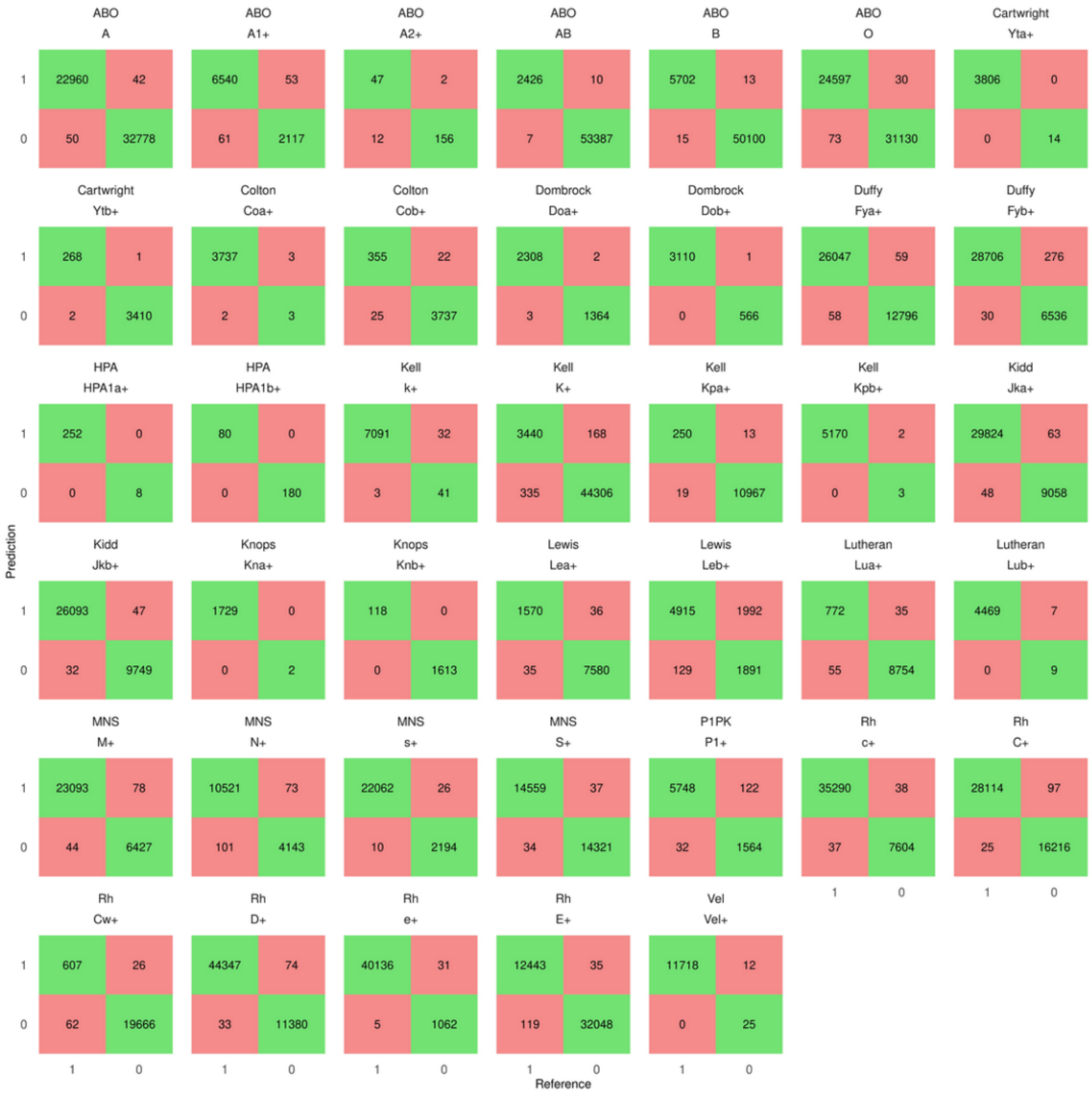

Fig J. Confusion matrices for the Danish random forest models in the Danish train data set

Confusion matrices for the Danish antigen classification models in the Danish train data set are presented in alphabetical order of the blood group systems. The RBC antigen/phenotype and HPA-1 typing results are on the x-axis and the model predictions on the y-axis. The antigen-negative samples are denoted by 0 and the antigen-positive samples by 1 on both axes. The numbers of true positive and true negative samples are depicted in the green boxes and the numbers of false positive and false negative samples in the red boxes.

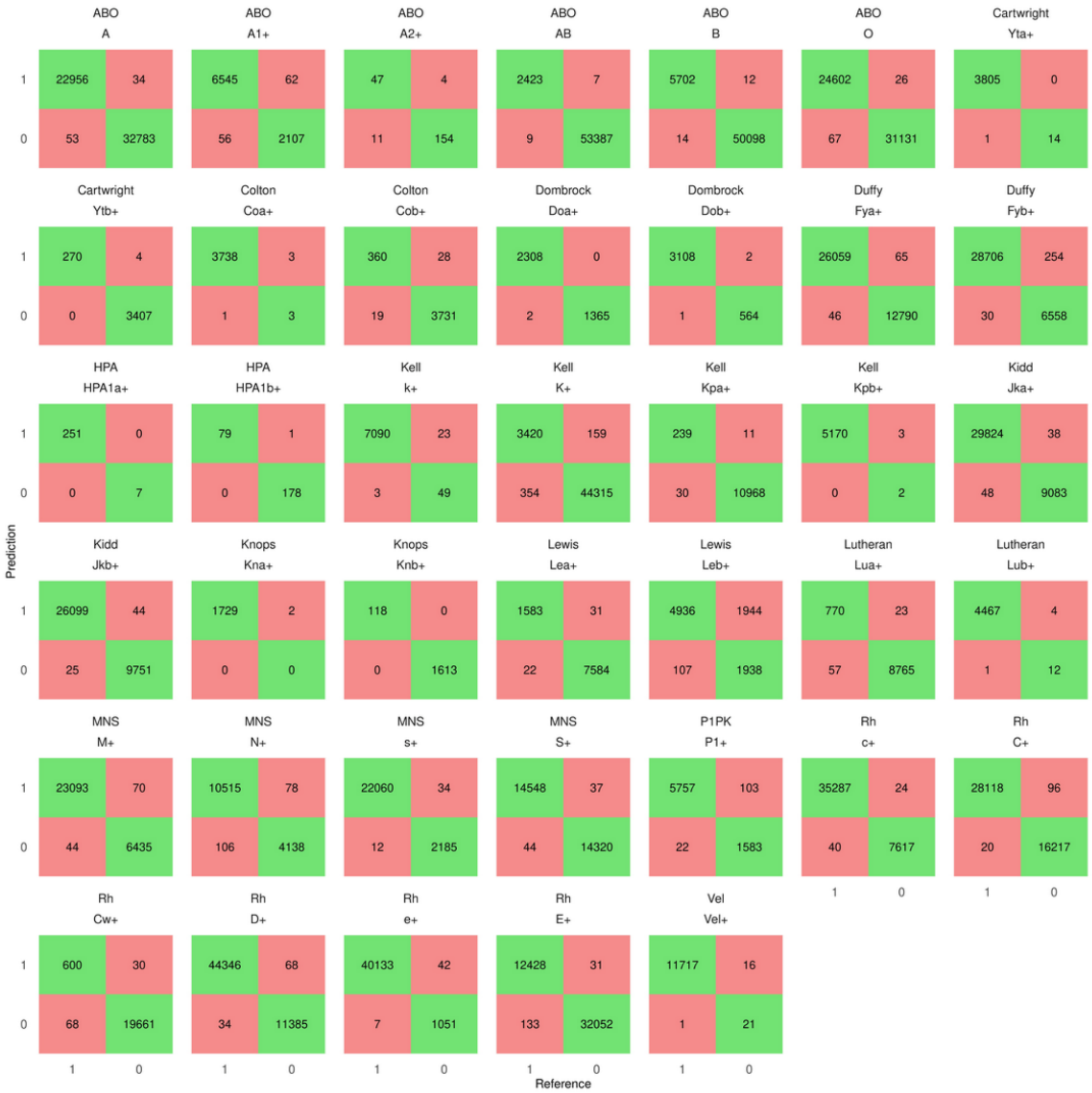

Fig K. Confusion matrices for the Danish random forest models in the Danish test data set

Confusion matrices for the Danish antigen classification models in the Danish test data set are presented in alphabetical order of the blood group systems. The RBC antigen/phenotype and HPA-1 typing results are on the x-axis and the model predictions on the y-axis. The antigen-negative samples are denoted by 0 and the antigen-positive samples by 1 on both axes. The numbers of true positive and true negative samples are depicted in the green boxes and the numbers of false positive and false negative samples in the red boxes.

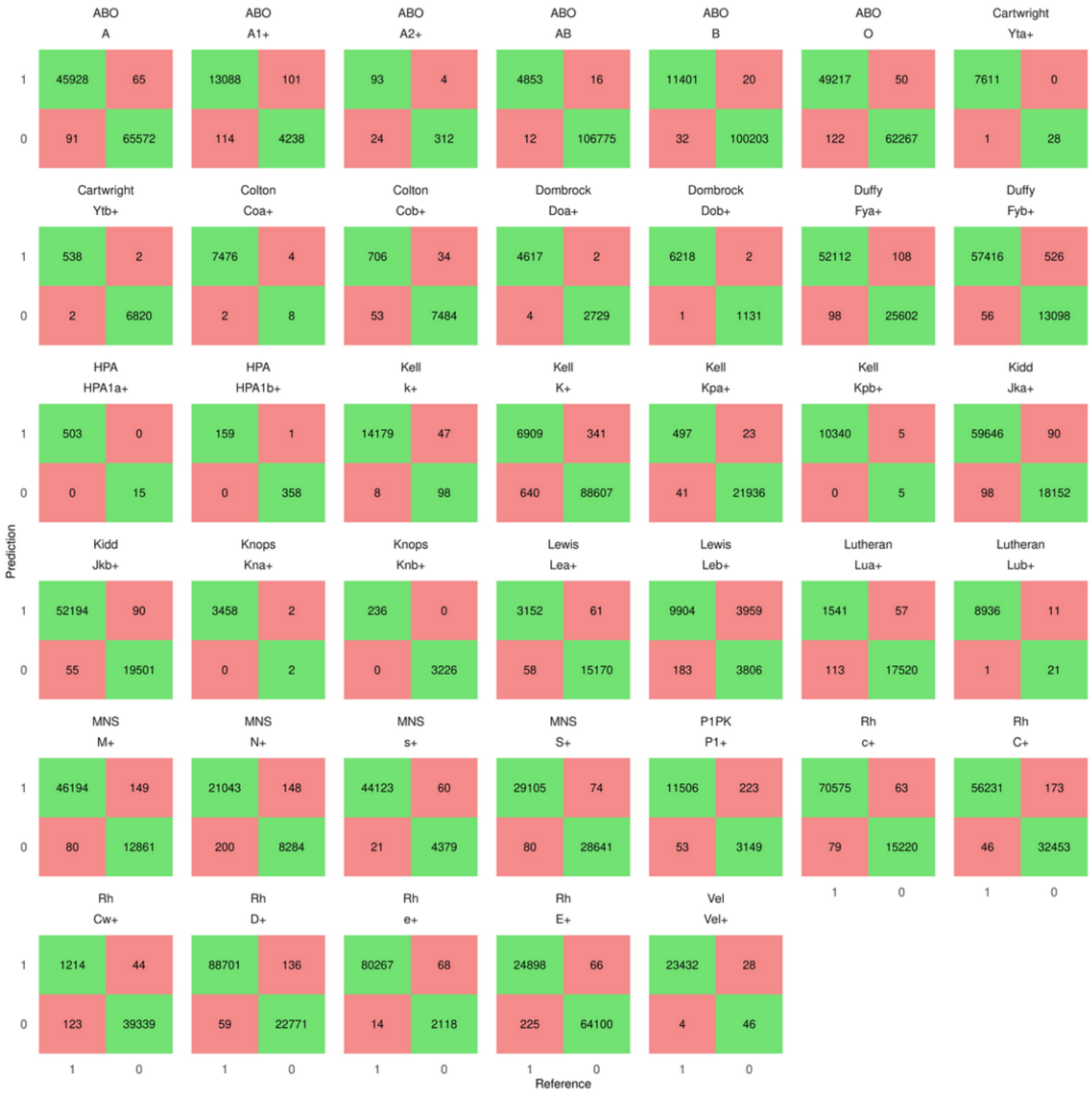

Fig L. Confusion matrices for the Danish random forest models in the Danish full data set

Confusion matrices for the Danish antigen classification models in the Danish full data set are presented in alphabetical order of the blood group systems. The RBC antigen/phenotype and HPA-1 typing results are on the x-axis and the model predictions on the y-axis. The antigen-negative samples are denoted by 0 and the antigen-positive samples by 1 on both axes. The numbers of true positive and true negative samples are depicted in the green boxes and the numbers of false positive and false negative samples in the red boxes.
