# Supplementary material for: A machine-learning method for biobank-scale genetic prediction of blood group antigens
Source: PLoS Comput Biol. 2024 Mar 21;20(3):e1011977. doi: 10.1371/journal.pcbi.1011977 (PMC10986993; doi:10.1371/journal.pcbi.1011977)
Supplement: Attachment — Submitted filename: Response to reviewers.pdf [file pcbi.1011977.s004.pdf]

Response to Reviewers

We thank the Reviewers for all the valuable comments and for the opportunity to revise our submission PCOMPBIOL-D-23-01617. Please find our detailed responses in red below. Line numbers refer the marked-up version of the revised manuscript.

Sincerely,
Kati Hyvärinen, PhD
Corresponding author

Comments to the Authors:
Please note here if the review is uploaded as an attachment.
Response: A marked-up copy of the revised manuscript, a clean copy of the revised manuscript and updated figures and Supplementary material are uploaded as attachments.

Reviewer #1: This manuscript introduces a novel random forest classification approach, designed as a stand-alone and freely accessible tool, for the probabilistic determination of red blood cell (RBC) antigens and HPA-1 from genotyping data obtained via array technology. The results demonstrate high overall accuracy, and the method is suitable for biobank-scale research and screening of antigen-negative donors. In reviewing this manuscript, I appreciate its contributions to the field; however, I have several concerns that, if addressed, could strengthen the paper and its clarity:

1.For the computational model presented, it would be beneficial for the manuscript to articulate more clearly the model's inputs and outputs in the Methods section. A detailed description of what data the model requires and what results it produces will aid in understanding and potentially increase the manuscript's appeal to a broader audience.

Response: We thank the Reviewer for the helpful suggestion. The input and output of the model fitting are now presented in the new Figure 3 and the input and output of the applying the Finnish models in the new Figure 4. The input of the model is imputed genotype data in chromosomal variant call format (or PLINK) and antigen data in text format (e.g., Kpa+/Kpa-). This information has now been added to the Methods section lines 369–375 and 433–438 in the marked-up version of the revised manuscript.

2.While the random forest model has been emphasized, it would be valuable to see a comparison with other models, including different ensemble models or neural network models.

Response: To compare our random forest classification approach with a different ensemble model, the prediction of RBC and HPA-1 antigens was performed using gradient boosting method. Information has been added to new Figure 2B, the Methods section lines 400–411, the Results section lines 159–166, and Discussion section lines 282–286 in the marked-up version of the revised manuscript.

Neural network models can handle missing input data problem but parameter tuning benefit from large train set. In our case, we did not have large train set at our disposal and had only limited data from rare antigen types available. Therefore, we adopted a modelling approach able to efficiently handle this kind of data. This information has been added to the Discussion section lines 286–294 in the marked-up version of the revised manuscript.

3.Given that variational neural network models have been shown to handle or impute missing values effectively in feature space, it would be interesting to explore or discuss whether such models could be applicable or advantageous in this context.

Response: We agree with the Reviewer that accurate imputation of missing genotype data would be advantageous in optimizing model performance. Variational neural network models are lucrative option for large data sets with missing genotype data. However, if the input data is relatively small, using specialized tools designed for imputing missing genotypes independently of input data size prior to modelling are a superior option to neural networks requiring large data sets. The most effective imputation of input genotypes is achieved using a population-specific reference panel. This topic has now been added to the Discussion section lines 286–294 in the marked-up version of the revised manuscript.

4.The manuscript could benefit from a more streamlined presentation of the figures in the main text. Reducing the number of sub-images or focusing on the most illustrative ones could enhance the clarity and conciseness of the paper.

Response: We thank the Reviewer for the constructive suggestion. The figures presenting confusion matrices and posterior probability boxplots separately for models have now been moved to supplementary material. The new Figure 2 presents the accuracy metrics in a more streamlined fashion. ROC and precision-recall curves combining prediction results from all the Finnish test data are presented in Supplementary Figure 4.

Addressing these points would not only improve the clarity of the manuscript but also expand its depth and comparative context, potentially making it a more comprehensive and valuable resource for the field.

Reviewer #2: Overall, this paper does an excellent job. This paper effectively demonstrates the feasibility and high accuracy of using computational methods for determining RBC antigens and HPA-1 from genotyping array data. The rigorous testing and validation across Finnish and Danish cohorts underscore the robustness of the proposed models. Furthermore, the high balanced accuracy rates achieved highlight the potential of these models for large-scale applications in blood transfusion and genomics research. The paper's clear presentation of methodologies and findings significantly contributes to advancing precision medicine in the field of blood transfusion

I have a few comments:

1.Figure Resolution: The resolution of the figures is quite poor. Please verify if this is an issue with the review system.

Response: We agree with the Reviewer, the resolution of figures in the original submission PDF is insufficient. This could be an issue with the submission system. We have now upload our figure files to the Preflight Analysis and Conversion Engine (PACE) digital diagnostic tool, https://pacev2.apexcovantage.com. PACE should ensure that figures meet PLOS requirements and our figures have passed the test.

2. Model Analysis: I recommend including confusion matrices for the Finnish model, validated using Danish cohorts.

Response: Although we agree with the reviewer, unfortunately we are having major problems accessing the Danish sensitive individual-level data in the computing cluster. The antigen-level accuracy data is available in Supplementary Table 5.

3. Presentation of Accuracy Metrics: For tables like Table 2 and 4, consider using a boxplot or swarm plot for better clarity. Detailed tables could be moved to supplementary material to enhance readability.

Response: We thank the Reviewer for the constructive suggestion. Tables 2 and 4 have now been moved to supplementary material. The new Figure 2 presents the accuracy metrics in a more streamlined fashion. ROC and precision-recall curves combining prediction results from all the Finnish test data are presented in Supplementary Figure 4.

4. Interpretation of Data (Line 160): Describing 23 out of 40 as a 'majority' may not be accurate. Please reconsider this characterization.

Response: We have corrected “Majority” to “More than half” line 193 in the marked-up version of the revised manuscript.

5. Model Interpretation: Given the impressive performance, employing explainable methods, such as feature importance or Shapley values, for interpreting the model is suggested.

Response: The importance values for the genomic variants are presented in the files “Supplementary Data 2 Finnish model important variables” and “Supplementary Data 3 Danish model important variables” in the Importance column. This information has been added to the lines 396 and 451 in the marked-up version of the revised manuscript.

Reviewer #3: In this manuscript, Hyvärinen et al. adopted random forest models for determining compatibility of the patient and donor red blood cell (RBC) antigens from genotyping array data. By testing on Finnish Blood Service and validating on a Danish blood cohort. The manuscript is organized and relatively easy to read.

I have 5 major concerns.

1 Can you add a schematic figure to explain the input and output of the model? It is not clear to me what is the dimension of the input genotype data and what is the representation of antigen. Is it just one-hot encoding?

Response: We thank the Reviewer for the helpful suggestion. The input and output of the model fitting are now presented in the new Figure 3 and the input and output of the applying the Finnish models in the new Figure 4. The input of the model is imputed genotype data in chromosomal variant call format (or PLINK) and antigen data in text format (e.g., Kpa+/Kpa-). This information has now been added to the Methods section lines 369–375 and 433–438 in the marked-up version of the revised manuscript.

2 Have the authors considered discussing the potential use cases of these models in other biobank data?

Response: Our method is suitable for initial screening for antigen-negative donors at biobank-scale in blood donor biobanks. Additionally, successful prediction of polygenic blood groups may facilitate the research of disease associations in other large biobanks containing different ethnic and phenotype data. This topic has now been discussed the Discussion section lines 307–309 and 318–322 in the marked-up version of the revised manuscript.

3 Are there some competing methods for the same tasks? Have the authors considered other ML models such as XGBoosting?

Response: As the Reviewer points out, several ML models could be utilized in prediction. To compare our random forest classification approach with a different ensemble model, the prediction of RBC and HPA-1 antigens was performed using gradient boosting method. Information has been added to new Figure 2B, the Methods section lines 400–411, the Results section lines 159–166, and Discussion section lines 282–285 in the marked-up version of the revised manuscript.

Neural network models can handle missing input data problem but parameter tuning benefit from large train set. In our case, we did not have large train set at our disposal and had only limited data from rare antigen types available. Therefore, we adopted a modelling approach able to efficiently handle this kind of data. This information has been added to the Discussion section lines 285-294 in the marked-up version of the revised manuscript.

4 It would be more intuitive to show AUROC curve and precision-recall curve.

Response: ROC and precision-recall curves combining prediction results from all the Finnish test data are now presented in Supplementary Figure 4.

5 The visualization and table need to be greatly improved. All models seem performing reasonably well. A lot of results can be put into supplementary results. The authors need to think carefully how to summarize the results for the audience.

Response: We thank the Reviewer for the constructive suggestion. The figures presenting confusion matrices and posterior probability boxplots separately for models have now been moved to supplementary material. In addition, Tables 2 and 4 have now been moved to supplementary material. The new Figure 2 presents the accuracy metrics in a more streamlined fashion.

I have 1 minor comment.
The authors validate using the Danish cohort. Have the authors considered validating the Africa/Asia cohort?

Response: We agree with the reviewer that it would be very important to validate the method and models in non-European populations. We have added this to Discussion section lines 307–309 in the markup version of the revised manuscript. However, to our knowledge there is no suitable phenotyped data publicly available.
